# Growth factor progranulin promotes tumorigenesis of cervical cancer via PI3K/Akt/mTOR signaling pathway

**DOI:** 10.18632/oncotarget.11126

**Published:** 2016-08-09

**Authors:** Tingting Feng, Lin Zheng, Feng Liu, Xiaoying Xu, Sheng Mao, Xiao Wang, Juan Liu, Yi Lu, Weiming Zhao, Xiuping Yu, Wei Tang

**Affiliations:** ^1^ Department of Pathogenic Biology, Shandong University School of Medicine, Jinan, Shandong, China; ^2^ Department of Biochemistry and Molecular Biology, Shandong University School of Medicine, Jinan, Shandong, China; ^3^ Department of Pathology, Shandong University School of Medicine, Jinan, Shandong, China; ^4^ Microbiological Laboratory, The Affiliated Hospital of School of Medicine of Ningbo University, Ningbo, Zhejiang, China

**Keywords:** progranulin, mTOR signaling, tumorigenesis, transformation, cervical cancer

## Abstract

Progranulin (PGRN) is an autocrine growth factor with tumorigenic roles in various tumors including cervical cancer. In this study, we investigated mammalian target of rapamycin (mTOR) signaling in response to PGRN induction and the contribution of the PGRN-stimulated PI3K/Akt/mTOR signaling pathway in the transformation and progression of cervical cancer. Here we identified a strong linkage between PGRN and phosphorylated-mTOR in cervical cancer tissues. PGRN promoted the phosphorylation of mTOR and activated mTOR signaling in human cervical mucosa epithelial cells and cervical cancer cells, and TNFR2 was needed for PGRN-stimulated mTOR signaling. Inhibition of mTOR signaling with rapamycin decreased PGRN-stimulated protein synthesis, transformation and proliferation of cervical cells *in vitro*, and tumor formation and growth *in vivo*. Thus, our findings update the signal transduction pathways of PGRN by suggesting that mTOR signaling contributes to PGRN-stimulated carcinogenesis of cervical cancer. Inhibition of PGRN/PI3K/Akt/mTOR signaling may be targeted in treatment of cervical cancer.

## INTRODUCTION

Cervical cancer is the fourth death cause of female cancer worldwide, with an estimated 527,624 new cases and 265,653 deaths in 2012, and the second most common female cancer and cause of cancer deaths in women aged 15-44 years in the world [1].

Progranulin (PGRN), also known as acrogranin, proepithelin, or GP88/PC cell-derived growth factor, is an autocrine growth factor with multiple functions that has been implicated in various physiologic and disease processes [2, 3]. PGRN was originally identified as a growth factor for cancer cells and is strongly believed to mediate tumorigenesis in tumors including breast, ovarian, prostate, bladder, and liver cancer [4, 5]. Our previous study first demonstrated that PGRN is overexpressed in cervical cancer cells and tissues and contributes to cervical cancer tumorigenesis *in vitro* and *in vivo* [6].

PGRN stimulates Shc and p44/42 mitogen-activated protein kinase in the extracellular-regulated kinase (Erk) pathway and phosphatidylinositol 3-kinase (PI3K), protein kinase B/Akt, and p70S6 kinase in the PI3K pathway [4], which are essential for PGRN-mediated cell division, survival and invasion [7]. Mammalian target of rapamycin (mTOR) Ser/Thr kinase is a member of the PI3K-like kinase family and is activated by phosphorylation at Ser2448 by Akt via PI3K/Akt signaling and by autophosphorylation at Ser2481 [8, 9].

As the catalytic subunit, mTOR participates in two different complexes, rapamycin-sensitive mTORC1 and rapamycin-insensitive mTORC2 [10, 11], which have distinct physiological functions and are regulated differently. mTOR is a critical kinase for regulation of many cellular events, such as cell proliferation, growth, survival, differentiation, adhesion, motility, angiogenesis and metastasis [12–15]. Input from intracellular and extracellular cues, such as amino acids, stress, oxygen, energy, and growth factors, activate mTORC1 [16, 17]. The main downstream targets of mTORC1 include eIF4E binding protein 1 (4E-BP1) and 40S ribosomal protein S6 kinase (S6K), which regulate mRNA translation initiation and progression, thus controlling the rate of protein synthesis [18]. mTORC1 also controls lipogenesis and energy metabolism and inhibits autophagy and lysosome biogenesis, thereby promoting cell growth and proliferation [17]. Much less is known about the upstream activators of mTORC2. mTORC2 is considered to directly or indirectly respond to growth factors [17]. It phosphorylates and activates AGC kinase family members, including Akt, serum and glucocorticoid-induced protein kinase 1 (SGK1), and protein kinase C-α (PKCα), which regulate cell survival, cell cycle progression and metabolism as well as the cytoskeleton [19–22]. Abnormal activation of mTOR signaling occurs in various human cancers [11, 23, 24], including cervical cancer [25, 26].

PGRN stimulates PI3K/Akt, Erk signaling and phosphorylation of p70S6K at Thr389, the up- or downstream cascades of the mTOR signaling pathway, which implies that mTOR signaling may be involved in the intracellular signal transduction network of PGRN. Phosphorylation of p70S6K is increased in mouse embryo-derived 3T3-like R– cells when they overexpress PGRN [27]. As well, PGRN promotes myotube hypertrophy via the PI3K/Akt/mTOR pathway, as evidenced by PGRN stimulation of mTOR downstream factors phospho-Akt (Ser473), phospho-p70S6K (Thr389) and phospho-GSK-3α/β (Ser21/9) in C2C12 cells [28]. However, more detailed study of mTOR signaling in response to PGRN is needed, especially in cancer.

The main objective of this study was to obtain evidence of PGRN regulation of the mTOR signaling pathway and its contribution to PGRN-mediated transformation and progression of cervical cancer. We found level of PGRN correlated with that of phosphorylated mTOR in cervical cancer, and PGRN stimulated the phosphorylation of mTOR and activation of PI3K/Akt/mTOR signaling in cervical cells. Inhibition of mTOR signaling disrupted PGRN-stimulated protein synthesis, transformation and proliferation of cervical cells *in vitro* and tumor formation and growth in mice *in vivo*. Our findings reveal an essential pathological function of PGRN in regulating the mTOR signaling pathway in tumorigenesis of cervical cancer. The PGRN/PI3K/Akt/mTOR signaling pathway may be a novel candidate for targeted therapy in PGRN-associated malignancies, including cervical cancer.

## RESULTS

### Expression of PGRN increased with level of phosphorylated mTOR in cervical cancer tissues

**Figure 1:**
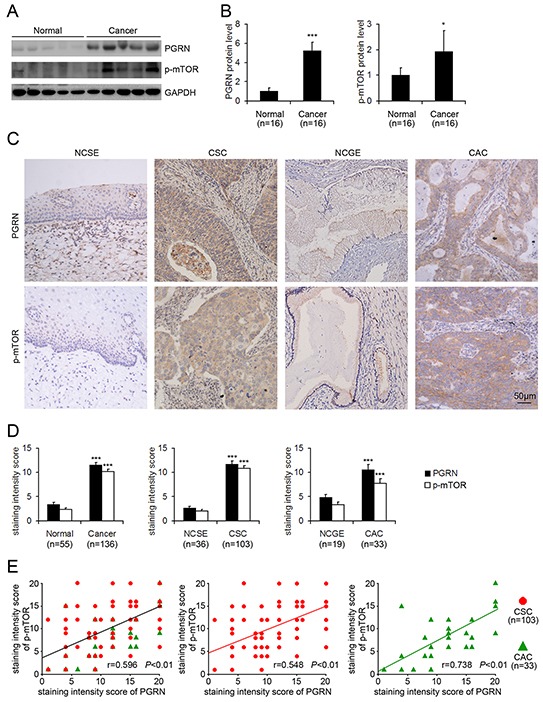
Increased protein level of progranulin (PGRN) and phospho-mTOR-Ser2448 and positive correlation in cervical cancer tissues Representative western blot gel **A.** and quantification **B.** showing the protein levels of PGRN and phospho-mTOR-Ser2448 in normal cervical tissues (Normal) and cervical cancer tissues (Cancer). GAPDH was a loading control. Data are mean ± SD. *, P<0.05; ***, p<0.001 compared with normal cervical tissues. **C.** Representative photomicrographs of PGRN and phospho-mTOR-Ser2448 immunohistochemical staining in normal cervical squamous epithelium (NCSE), normal cervical glandular epithelium (NCGE), cervical squamous carcinoma (CSC) and cervical adenocarcinoma (CAC) samples. **D.** Quantification of staining intensity. Data are mean ± SEM. ***, p<0.001 compared with the normal cervical tissues, NCSE or NCGE. **E.** Pearson correlation analysis of PGRN expression and phosphorylated mTOR at Ser2448 in cervical cancer, cervical squamous carcinoma (CSC) and cervical adenocarcinoma (CAC).

Western blot assay showed that the protein level of PGRN and phosphorylation of mTOR at Ser2448 were increased in cervical cancer tissues compared with normal cervical tissues (Figure 1A and 1B). Immunohistochemistry revealed greater PGRN immunoreactivity in human cervical cancer than normal cervix samples (Figure 1C and 1D). The protein level of PGRN was greatly increased in cervical squamous carcinoma (CSC) and cervical adenocarcinoma (CAC) tissue as compared with normal cervical squamous epithelium (NCSE) and normal cervical glandular epithelium (NCGE), respectively (Figure 1C and 1D). In addition, the expression of PGRN in the edge was higher as compared with that in the center of CSC cancer nests (Supplementary Figure S1). Importantly, the staining intensity scores of PGRN and phosphorylated mTOR at Ser2448 in cervical cancer, CSC and CAC, were positively correlated (Figure 1E), which suggests that cancer-associated activation of mTOR signaling may be involved in PGRN stimulation during the progression of cervical cancer.

### PGRN stimulated the phosphorylation of mTOR in cervical cells

**Figure 2:**
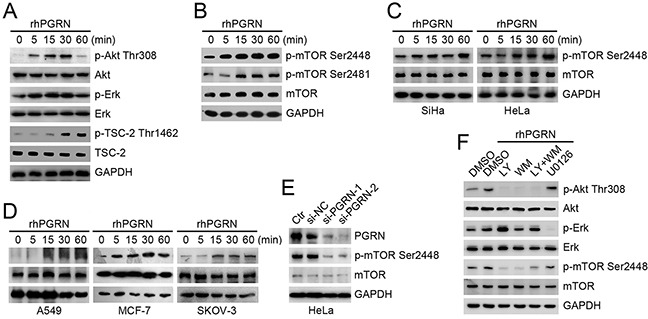
PGRN promoted phosphorylation of mTOR, which depended on PI3K/Akt signaling Western blot assay of phospho-Akt-Thr308, phospho-Erk-Thr202/Tyr204 and phospho-TSC-2-Thr1462 **A.** and phospho-mTOR-Ser2448 and Ser2481 **B.** in H8 cells with 500 ng/mL rhPGRN treatment. Western blot assays of phospho-mTOR-Ser2448 in **C.** SiHa, HeLa, **D.** A549, MCF-7 and SKOV-3 cells with rhPGRN treatment. **E.** Level of phospho-mTOR at Ser2448 in HeLa cells transfected with negative control siRNA (si-NC) and PGRN siRNA (si-PGRN). Ctr was parent HeLa cells. **F.** Western blot assay of mTOR activation in cervical cells stimulated with rhPGRN alone or with inhibitors of PI3K/Akt or MEK/Erk signaling pathways. GAPDH was a loading control.

To determine whether PGRN can activate mTOR signaling, we first detected the status of the upstream modulators Akt, Erk, and tuberous sclerosis 2 (TSC-2). The levels of phosphorylated Akt-Thr308 and Erk were elevated, and phosphorylation of TSC-2 at Thr1462, an Akt-targeting site, was strongly induced in human cervical mucosa epithelial H8 cells with recombinant human PGRN (rhPGRN) treatment (Figure 2A). We next investigated the phosphorylation of mTOR with rhPGRN treatment. rhPGRN markedly enhanced the phosphorylation of mTOR at Ser2448 and Ser2481 in H8 cells (Figure 2B). rhPGRN-treated cervical cancer SiHa and HeLa cells also showed increased phosphorylation of mTOR at Ser2448 (Figure 2C). We also found that rhPGRN treatment enhanced the phosphorylation of mTOR at Ser2448 in HeLa cells within 24 h (Supplementary Figure S2A). PGRN-activated mTOR was confirmed in other cancer cells, including A549, MCF-7 and SKOV-3 cell lines (Figure 2D).

To determine whether a specific level of PGRN is required for orchestrating the activation of mTOR in cervical cells, we suppressed PGRN gene expression in HeLa cells by an siRNA approach (Figure 2E). Reduced expression of PGRN in HeLa cells decreased the phosphorylation of mTOR at Ser2448 (Figure 2E). We further evaluated the dependence of mTOR phosphorylation on PGRN signaling cascades. H8 cells pretreated with inhibitors of PI3K/Akt or MEK/Erk signaling were exposed to rhPGRN, and phosphorylation of mTOR was examined. PGRN-stimulated mTOR phosphorylation was almost completely blocked by inhibitors of PI3K/Akt, LY294002 and/or wortmannin (Figure 2F). Pretreatment with inhibitor of MEK/Erk, U0126, partially attenuated PGRN-stimulated mTOR phosphorylation in H8 cells (Figure 2F), which was also confirmed in cervical cancer HeLa cells (Supplementary Figure S2B).

### PGRN promoted activation of mTORC1 and mTORC2 in cervical cells

**Figure 3:**
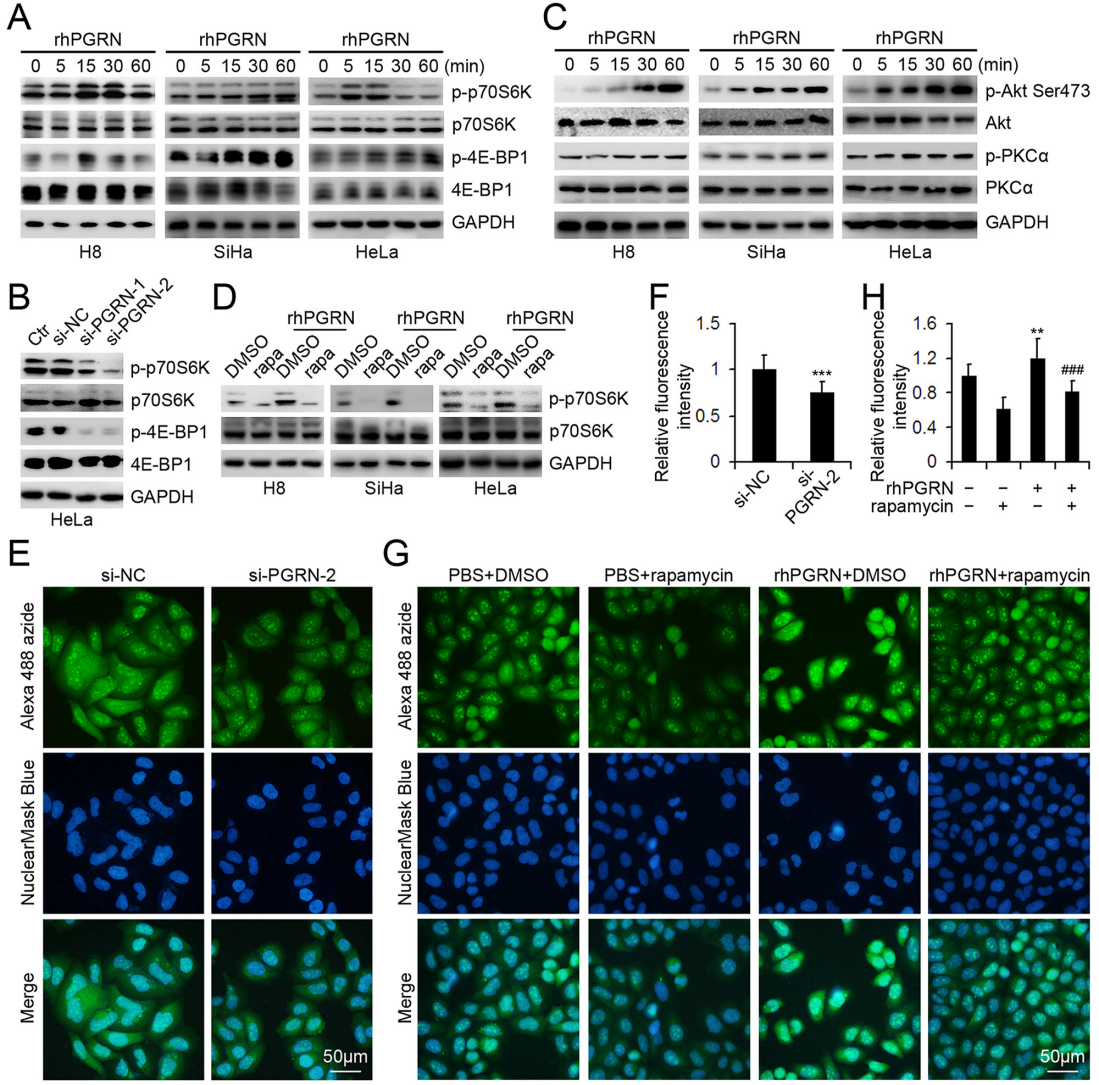
PGRN regulated the phosphorylation of mTORC1 and mTORC2 downstream molecules and mTORC1-mediated protein synthesis **A.** Western blot assay of phospho-p70S6K-Thr389 and phospho-4E-BP1-Thr37/46 in cervical cells with 500 ng/mL rhPGRN treatment; **B.** phospho-p70S6K-Thr389 and phospho-4E-BP1-Thr37/46 in HeLa cells transfected with PGRN siRNA (si-PGRN) or control siRNA (si-CTR); **C.** phospho-Akt-Ser473 and phospho--PKCα-Thr638 in cervical cells with 500 ng/mL rhPGRN treatment; and **D.** mTORC1 activation in cervical cells stimulated with 500 ng/mL rhPGRN alone or with 100 μM rapamycin. GAPDH was a loading control. Representative micrographs **E.** and quantification of Alexa 488 fluorescence intensity **F.** of HeLa cells transfected with si-NC or si-PGRN-2 treated with O-propargyl-puromycin (OPP). Representative micrographs **G.** and quantification of Alexa 488 fluorescence intensity **H.** of HeLa cells treated with PBS or rhPGRN in the absence or presence of rapamycin followed by OPP treatment. Nuclei were revealed by HCS NuclearMaskBlue Staining. The relative fluorescence intensity was determined by setting the fluorescence intensity of HeLa cells transfected with si-NC or treated with PBS + DMSO to 1. Data are mean ± SD. **, P<0.01; ***, p<0.001 compared with HeLa cells transfected with si-NC or treated with PBS + DMSO. ###, p<0.001 compared with HeLa cells treated with rhPGRN + DMSO.

We assessed the PGRN-regulated phosphorylation of downstream molecules in mTORC1 signaling in cervical cells. rhPGRN stimulation enhanced the levels of phospho-p70S6K at Thr389 and phospho-4E-BP1 at Thr37/46 in H8, SiHa, and HeLa cells (Figure 3A). In addition, HeLa cells transfected with siRNA targeting PGRN (si-PGRN) showed decreased p70S6K and 4E-BP1 phosphorylation as compared with the control (si-CTR) and parent cells (Figure 3B). rhPGRN enhanced levels of mTORC2 downstream molecules, such as phospho-Akt at Ser473 and phospho-PKCα at Thr638 (Figure 3C).

We next examined the activation of mTORC1 in rhPGRN-stimulated cervical cells in the presence of rapamycin, an inhibitor of mTOR signaling pathway. Rapamycin blocked PGRN-stimulated phosphorylation of p70S6K in H8, SiHa and HeLa cells, so rapamycin attenuated PGRN-activated mTORC1 (Figure 3D). Given that mTORC1 controls the rate of protein synthesis, we suppressed PGRN expression with siRNA and found that the newly synthesized protein levels in HeLa cells were reduced (Figure 3E and 3F). rhPGRN enhanced protein synthesis in HeLa cells, which was abolished by rapamycin pretreatment (Figure 3G and 3H).

### Reduced expression of TNFR2 impaired PGRN-stimulated mTOR signaling in cervical cancer cells

**Figure 4:**
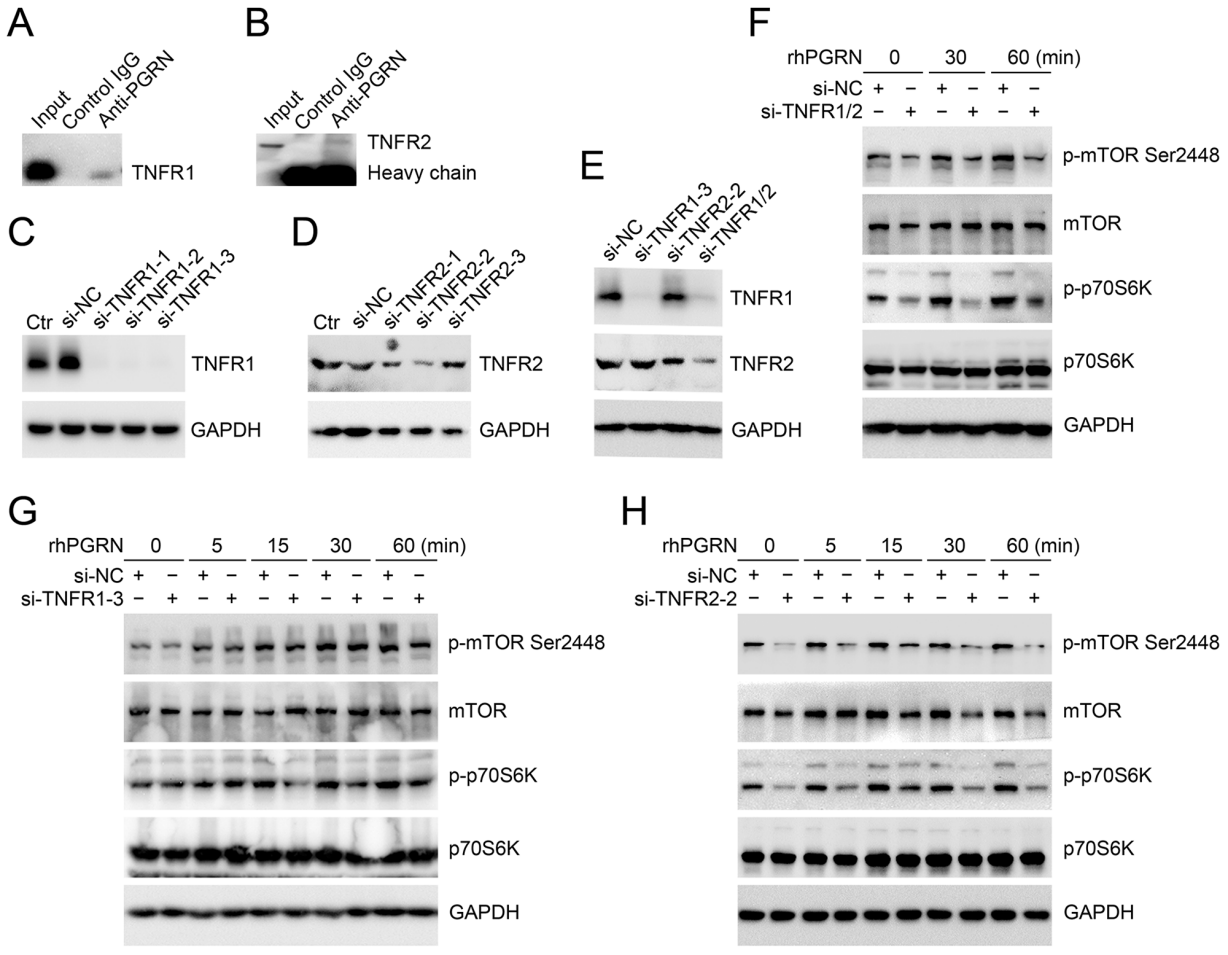
TNFR2 was needed for PGRN-stimulated mTOR signaling in cervical cancer cells Co-IP assays for PGRN binding with TNFR1 **A.** and TNFR2 **B.** Level of TNFR1 or/and TNFR2 in HeLa cells transfected with negative control siRNA (si-NC), TNFR1 siRNA (si-TNFR1) **C.** TNFR2 siRNA (si-TNFR2) **D.** and si-TNFR1 and si-TNFR2 **E.** Ctr was parent HeLa cells. Western blot assay of phospho-mTOR-Ser2448 and phospho-p70S6K-Thr389 in si-TNFR1 and si-TNFR2 **F.** si-TNFR1 **G.** or TNFR2 **H.** transfected HeLa cells with 500 ng/mL rhPGRN treatment. GAPDH was a loading control.

Tumor necrosis factor receptors (TNFRs) are potential binding receptors of PGRN. The binding of PGRN with TNFR1 and TNFR2 in HeLa cells was confirmed by Co-immunoprecipitation (Co-IP) assays (Figure 4A, 4B). To investigate whether TNFRs are needed for PGRN-mediated mTOR signaling, we detected the phosphorylation of mTOR and p70S6K in cervical cancer cells with reduced expression of TNFR1 or/and TNFR2 after rhPGRN treatment. The expression of TNFR1 or/and TNFR2 in HeLa cells was suppressed by transfection of si-TNFR1 or/and si-TNFR2 (Figure 4C–4E). As shown in Figure 4F, reduced expression of TNFR1 and TNFR2 impaired rhPGRN-stimulated phosphorylation of mTOR and p70S6K. Interestingly, reduced expression of TNFR2 but not TNFR1 in HeLa cells dramatically blocked rhPGRN-stimulated mTOR signaling (Figure 4G, 4H).

### Inhibition of mTOR signaling disrupted PGRN-stimulated transformation of non-malignant cervical cells

**Figure 5:**
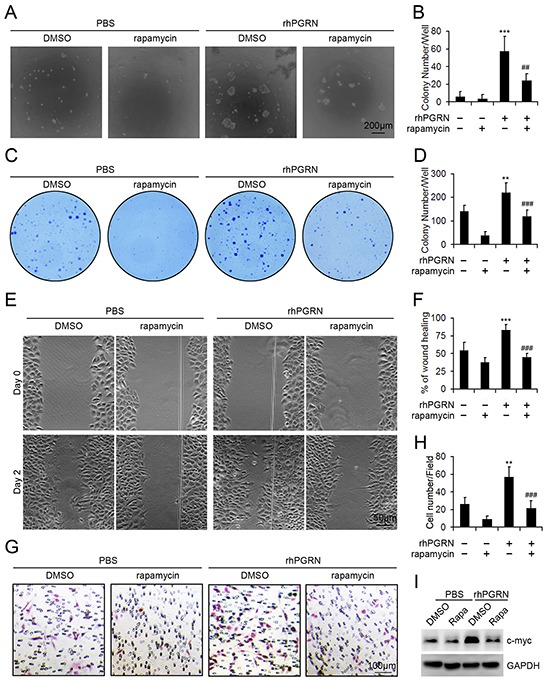
Rapamycin inhibited the transformation of H8 cells treated with rhPGRN Representative micrographs **A.** and colony counting **B.** of colony formation assay in soft agar culture of H8 cells treated with PBS or 500 ng/mL rhPGRN in the presence or absence of 100 μM rapamycin. Representative photographs **C.** and colony counting **D.** of H8 cells in medium with 1% FBS treated with rhPGRN in the presence or absence of rapamycin. **E.** Wound healing assay of H8 cells treated with rhPGRN in the presence or absence of rapamycin. **F.** Percentage of wound healing. The normalized values were calibrated against the wound widths at day 0 that were arbitrarily set to 100%. **G.** Migration assay of H8 cells treated with rhPGRN in the presence or absence of rapamycin. **H.** Data analysis of migrated cells quantified by counting five fields under 20 × magnification. Data are mean ± SD. **, P<0.01; ***, p<0.001 compared with H8 cells treated with PBS + DMSO. ##, P<0.01; ###, p<0.001 compared with H8 cells treated with rhPGRN + DMSO. **I.** Western blot assay of c-myc in H8 cells treated with PBS or 500 ng/mL rhPGRN for 24 h after DMSO or 100 μM rapamycin pretreatment. GAPDH was a loading control.

Transformed cells have abnormal growth parameters and behaviors, such as reduced requirement for serum growth factors, anchorage independent growth, and tumorogenic ability when transplanted into animals. In cell transformation analysis, rhPGRN treatment enhanced the colony-formation ability of non-transformed H8 cells in soft-agar medium, and PGRN-supported anchorage independent growth was decreased with rapamycin pretreatment (Figure 5A, 5B). rhPGRN treatment enhanced the survival of H8 cells under low serum cultivation, which indicates reduced requirement for serum growth factors (Figure 5C, 5D). Inhibition of mTOR signaling reduced the survival of H8 cells even in the presence of rhPGRN (Figure 5C, 5D). We also examined the effect of PGRN-stimulated mTOR signaling on the motility of cells by monolayer wound assay (Figure 5E). In the absence of rapamycin, wound closure was greater in H8 cells with than without rhPGRN (Figure 5F). Exposure to rapamycin with rhPGRN reduced the wound closure of H8 cells (Figure 5F). Furthermore, inhibition of mTOR signaling disrupted rhPGRN-promoted migration of H8 cells (Figure 5G, 5H). In addition, rhPGRN increased the level of transformation-associated c-myc in H8 cells, which was diminished by rapamycin pretreatment (Figure 5I).

### Rapamycin blocked PGRN-stimulated proliferation of cervical cells

**Figure 6:**
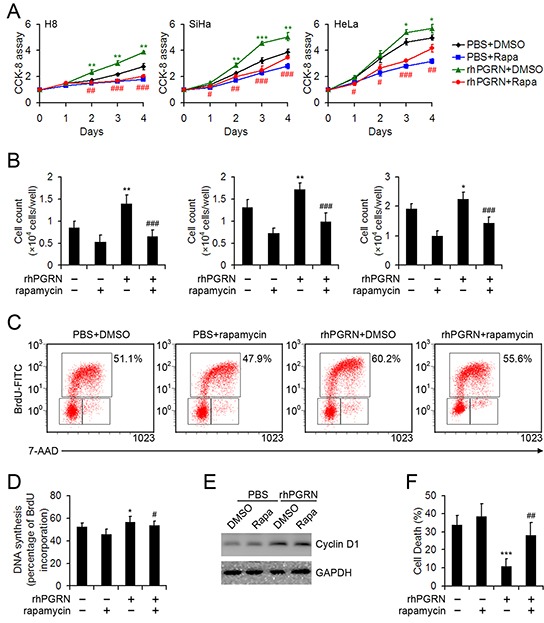
Rapamycin inhibited the proliferation-promoting and anti-anoikis role of PGRN in cervical cells Cell proliferation from day 0-4 **A.** and cell counting at day 3 **B.** for H8, SiHa and HeLa cells treated with PBS or 500 ng/mL rhPGRN in the presence or absence of 100 μM rapamycin. Representative flow cytometry **C.** and quantification of the proportion of BrdU^hi^ PI^low^ cells representing DNA synthesis **D.** of HeLa cells treated with PBS or 500 ng/mL rhPGRN for 6 h after DMSO or 100 μM rapamycin measured by BrdU incorporation. **E.** Western blot assay of cyclin D1 in HeLa cells treated with PBS or 500 ng/mL rhPGRN for 24 h after DMSO or 100 μM rapamycin pretreatment. GAPDH was a loading control. **F.** Quantification of the effect of PGRN and rapamycin on anoikis in HeLa cells induced by detachment. Data are mean ± SD. *, P<0.05; **, P<0.01; ***, p<0.001 compared with cervical cells treated with PBS + DMSO. #, P<0.05; ##, P<0.01; ###, p<0.001 compared with cervical cells treated with rhPGRN + DMSO.

The proliferation-promoting role of PGRN in H8, SiHa, and HeLa cells was determined by CCK-8 and cell counting assays; pretreatment with rapamycin inhibited PGRN-stimulated proliferation of cervical cells (Figure 6A, 6B). We compared DNA synthesis in HeLa cells with or without PGRN treatment in the presence or absence of rapamycin by using 5-bromo-2′-deoxyuridine (BrdU) incorporation assays. DNA synthesis was enhanced in PGRN-treated HeLa cells, as compared with PBS-treated cervical cancer cells; likewise, DNA synthesis was decreased when mTOR signaling was inhibited by rapamycin in cells (Figure 6C, 6D). PGRN-induced proliferation in cervical cells was at least in part due to activation of mTOR signaling. PGRN treatment increased the expression of cyclin D1, but the impact of rapamycin on cyclin D1 expression was limited (Figure 6E). In addition, PGRN treatment inhibited detachment-induced anoikis of HeLa cells, which was disrupted by inhibition of mTOR signaling (Figure 6F).

### Rapamycin inhibited PGRN-stimulated tumor formation and growth in nude mice

**Figure 7:**
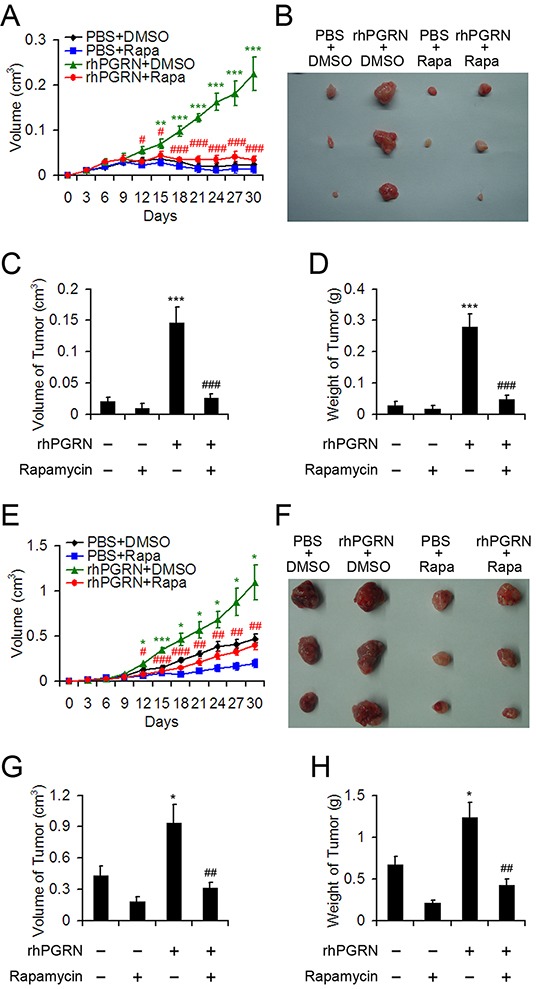
Rapamycin disrupted rhPGRN-induced tumor formation and growth in nude mice implanted with H8 or HeLa cells Tumor growth curves **A.** and photographs **B.** of tumors with H8 cell implantation and treatment with PBS or rhPGRN concomitant with DMSO or rapamycin. Volume **C.** and weight **D.** of tumors from mice with H8 cell implantation and indicated treatment. Tumor growth curves **E.** and photographs **F.** of tumors with HeLa cell implantation and treated with PBS or rhPGRN concomitant with DMSO or rapamycin. Volume **G.** and weight **H.** of tumors from mice with HeLa cell implantation and indicated treatment. Data are mean ± SEM. *P< 0.05; **P< 0.01; ***P< 0.001 compared with PBS + DMSO. #P< 0.05; ## P< 0.01; ###P< 0.001 compared with rhPGRN + DMSO.

To investigate the biological consequence of PGRN-stimulated mTOR signaling in tumorigenesis of cervical cancer, we used nude mouse xenografts. H8 cells were injected into 6-week-old female nude mice. As expected, at 30 days after treatment, mice with H8 cell implantation barely developed tumor with PBS and DMSO or PBS and rapamycin, but tumor size was considerable with rhPGRN and DMSO (Figure 7A, 7B). However, rapamycin treatment almost completely blocked PGRN-stimulated tumor formation in H8-cell–injected mice (Figure 7A, 7B). The volume and weight of tumors measured at the end of experiment displayed the same tendency (Figure 7C, 7D), so mTOR signaling was critical for transformation action of PGRN in cervical carcinogenesis. In the tumor growth assay, HeLa cells were injected into nude mice, which were treated with PBS or rhPGRN in the absence or presence of rapamycin. rhPGRN increased the tumor growth in mice injected with HeLa cells (Figure 7E–7H). The growth-promoting role of PGRN in tumors from mice with HeLa-cell implantation was inhibited with rapamycin treatment (Figure 7E–7H), so mTOR signaling was required for PGRN-stimulated progression of cervical cancer.

## DISCUSSION

In this study, we first report that PGRN levels were positively correlated with level of phosphorylated-mTOR in cervical cancer. A PGRN-stimulated mTOR signaling pathway was identified in non-transformed cervical mucosa epithelial cells and malignant cervical cancer cells. Inhibition of mTOR signaling with rapamycin decreased PGRN-mediated protein synthesis, transformation and proliferation of cervical cells *in vitro* and PGRN-stimulated tumor formation and growth in nude mouse xenografts. These data provide direct evidence for a PGRN/PI3K/Akt/mTOR signaling pathway contributing to tumorigenesis in cervical cancer and a potential target in its treatment.

The mTOR signaling plays a central role in cancer progression [29], and its activation has been observed in cervical cancer by immunocytochemical staining of certain components of mTOR signaling, such as upregulated expression of mTOR and p70S6K and elevated phosphorylation of mTOR, S6 and Akt-Ser473 in cervical cancer tissues [25, 26, 30, 31]. However, the activation status of mTOR (the catalytic subunit of mTORC1 and mTORC2) and upstream and downstream components of mTOR signaling in response to PGRN have not been well studied, especially in human cancer. Our data indicate a positive relation of PGRN level with level of phosphorylated mTOR in both CSC and CAC, which strongly suggests a potential relationship between PGRN and mTOR signaling in the progression of cervical cancer.

The major activated effector of mTOR signaling pathway is the serine/threonine protein kinase mTOR, which integrates a large panel of inputs, including nutrients and growth factors, and regulates protein synthesis and cell growth [32, 33]. In the present study, rhPGRN treatment altered the status of upstream components of mTORC1 in cervical mucosa epithelial cells, and enhanced the phosphorylation of mTOR at Ser2448 in cervical cancer and other cancer cell lines, suggesting PGRN-stimulated activation of mTOR signaling is a common phenomenon. Moreover, PGRN-stimulated phosphorylation of mTOR at Ser2448 was PI3K/Akt-dependent and partially MEK/Erk-dependent. The present study further demonstrated that PGRN stimulated mTORC1 activity in cervical cells. In rhPGRN-treated H8 cells, we observed phosphorylation of mTOR at Ser2481, which has been identified as a marker for intact mTORC2 signaling [34]. Phosphorylation of Akt at Ser473 and PKCα, the downstream substrates of mTORC2, was increased in cervical cells treated with rhPGRN. We previously reported that PGRN stimulates the phosphorylation and exclusion from the nucleus of forkhead box protein O1 (FoxO1) in cervical cells [6]. Given that mTORC2 is required for signaling for Akt-FoxO and PKCα [35], our data suggest that PGRN, as a growth factor, is also a stimulator for activation of mTORC2 in cervical cells.

It is previously reported that PGRN binds to TNFRs and has therapeutic effects in inflammatory arthritis [36]. This finding stimulated the explorations of PGRN/TNFR in various diseases and conditions [37]. Based on the confirmation of PGRN binding to TNFR1 and TNFR2 in HeLa cells, we demonstrated that TNFR2 was needed for PGRN-stimulated mTOR signaling in cervical cancer cells. Although, our data suggested TNFR2 is a key component in PGRN/mTOR signaling, other mechanism may also contribute to the regulation of this signaling and the exact and detailed transduction pathways still need further study.

Inhibition of mTOR by rapamycin and its analogs appears to be efficient in cancer therapies [43]. Rapamycin administration caused a remarkable decrease in tumor burden in nude mice with implantation of cervical cancer cells [31]. Therapy targeting PI3K/Akt/mTOR signaling has shown meaningful clinical benefits in cervical cancer [44]. The present study showed that PGRN contributed to the malignancy of cervical cancer, and inhibition of mTOR signaling by rapamycin disturbed PGRN-driven cervical cell transformation, proliferation and survival *in vitro* and tumor formation and growth *in vivo*. These data reveal that the PGRN/PI3K/Akt/mTOR signaling pathway contributes to the tumorigenesis of cervical cancer. The translation of a number of transformation-related proteins, such as cyclin D1, c-myc and vascular endothelial-growth factor, is thought to be increased by eIF4E released from the inhibition of 4E-BP1 with activation of mTOR signaling [40]. Rapamycin treatment inhibits translation of c-myc mRNA and S6K1 regulates the translation of c-myc via eIF4B [41, 42]. However, rapamycin only partially inhibits mTORC1 function, efficiently inhibiting S6K1 but not eIF4E [17], which may explain our finding of rapamycin inhibiting the protein level of only c-myc but not cyclin D1 with rhPGRN treatment. In addition, mTORC2 may directly drive tumorigenesis by activating Akt and SGK [17]. Further studies for the contribution of PGRN-stimulated mTORC2 in the transformation and progression of cervical cancer are needed depending on the generation of inhibitors specific for mTORC2.

**Figure 8:**
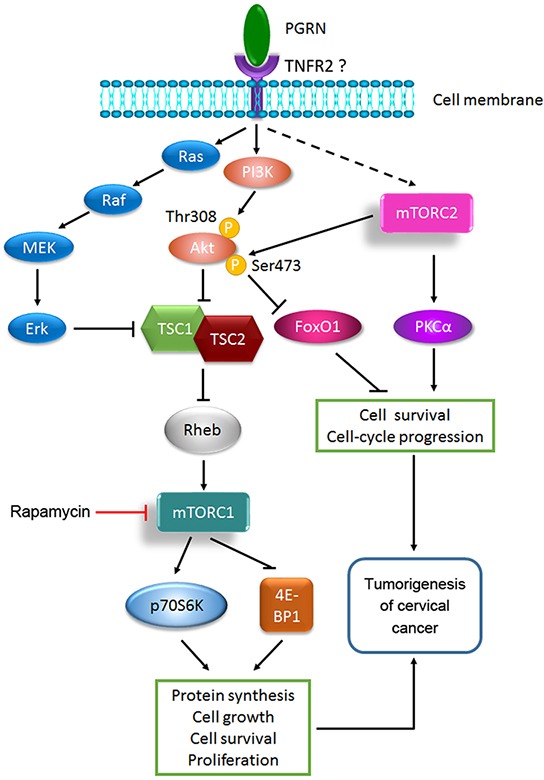
A proposed model of PGRN-stimulated mTOR signaling in the tumorigenesis of cervical cancer

On the basis of the present study and the various literature, a model was proposed for illustrating the role and regulation of PGRN in the tumorigenesis of cervical cancer (Figure 8). PGRN stimulates the activation of mTORC1 by PI3K/Akt and MEK/Erk signaling, and promotes the activation of mTORC2 by an unknown mechanism. In PGRN-stimulated mTOR signaling, PGRN stimulates the phosphorylation of mTOR in PI3K/Akt and partially MEK/Erk dependent manners. TNFR2 is needed for PGRN-stimulated mTOR signaling. PGRN promotes the tumorigenesis of cervical cancer by mTORC1- and mTORC2-enhanced protein synthesis, cell growth, survival, proliferation, and cell-cycle progression. Updating of PGRN-stimulated mTOR signaling can better our understanding of the critical role of PGRN in physiologic and disease processes but also provides new therapeutic interventions for PGRN in malignancies, including cervical cancer.

## MATERIALS AND METHODS

### Patients and tissue samples

We obtained 191 paraffin-embedded cervical tissue blocks, including 136 cervical cancer tissue (103 CSC and 33 CAC) and 55 normal cervical tissue (36 NCSE and 19 NCGE), and 16 paired frozen cancerous and matched adjacent normal cervical tissues from cervical cancer patients at Qilu Hospital, Shandong University, for immunohistochemistry staining and western blot assay respectively. We obtained patient consent for the use of these materials, and the study was approved by the Institutional Research Ethics Committee of Shandong University [no. 201401017]. Diagnoses followed the World Health Organization Classification of Tumors.

### Cell culture and treatment

All cells were cultured in DMEM (Invitrogen, Carlsbad, CA) containing 10% fetal bovine serum (FBS, Invitrogen), 100 IU/mL penicillin, 100 μg/mL streptomycin (Sigma-Aldrich, St. Louis, MO, USA), and 2 mmol/l L-glutamine at 37°C with 5% CO_2_ in a humidified incubator. The human cervical cancer cell lines SiHa and HeLa, human lung epithelial carcinoma cell line A549, human breast cancer cell line MCF-7, and human ovarian carcinoma cell Line SKOV-3 were purchased from the American Type Culture Collection (Manassas, VA). The HPV16 immortalized human cervical mucosa epithelial H8 cell line was obtained from the Department of Biophysics, Institute of Preclinical Medicine, Peking Union Medical University.

For signaling pathway analysis, cells were stimulated with 500 ng/mL recombinant human PGRN (rhPGRN, generated and purified as reported previously [45]) for the indicated times after serum-free treatment for 18 h. For inhibitor treatment, cells were incubated with dimethyl sulfoxide (DMSO), rapamycin (100 μM; Sigma-Aldrich, St. Louis, MO, USA), LY294002 (10 μM), wortmannin (1 μM), or U0126 (10 μM; all Cell Signaling Technology, Danvers, MA) containing medium for 1 hr before rhPGRN treatment.

### siRNA transfection

HeLa cells were seeded into 6-well culture plates at 1×10^5^ cells/well and cultured in medium without antibiotics. siRNA oligonucleotides (RiboBio, Guangzhou, China) were transfected into cells by the Lipofectamine 2000 reagent method (Invitrogen). The siRNA target sequences for human *GRN* were 5′-CTGTGTGTGACCTGATCCA-3′ and 5′-GTGAGCTGCCCAGATGGCT-3′, for human TNFR1 were 5′-CGGCATTATTGGAGTGAAA-3′, 5′-GAACCTACTTGTACAATGA-3′ and 5′-CTTGAAGG AACTACTACTA-3′, and for human TNFR2 were 5′-GGCTCAGAGAATACTATGA-3′, 5′-CCACAATG GGAGACACAGA-3′ and 5′-CATCAGACGTGGTGT GCAA-3′. Cells were harvested and used for experiments 48 h after transfection.

### Immunohistochemical staining

Immunoreactivity of PGRN and phosphorylated mTOR-Ser2448 in cervical tissue was assessed by immunohistochemistry as described [46] with mTOR (phospho-S2448) antibody (1:50, Abcam, UK) and human PGRN-specific antibody (1:100, Abcam). Semi-quantitative analysis of the staining intensity score was as described [47]. Briefly, semi-quantitative analysis was performed by three independent observers in a blinded fashion, using the following parameters: staining intensity was given a scale from 1 to 4 (1, no staining; 4, intense staining), and the abundance of positively stained cells was assessed on a score from 1 to 5 (1, no cells stained; 5, 100% stained). The staining intensity score was then calculated by multiplying the staining intensity by the staining abundance.

### Western blot assay

Total protein from cell cultures and cervical tissues was quantified by the Bradford assay. Western blot assay was performed as described [48] with the primary antibody for PGRN (1:1000, Abcam), phospho-Akt (Thr308, 1:1000), phospho-Akt (Ser473, 1:2000), total Akt (1:1000), phospho-Erk1/2 (Thr202/Tyr204, 1:2000) and total Erk1/2 (1:1000), phospho-TSC-2 (Thr1462, 1:1000), total TSC-2 (1:1000), phospho-mTOR-Ser2448 (1:1000), phospho-mTOR-Ser2481 (1:1000), total mTOR (1:1000), phospho-p70S6K (Thr389, 1:1000), total p70S6K (1:1000), phospho-4E-BP1 (Thr37/46, 1:1000), total 4E-BP1 (1:1000), phospho-PKCα (Thr638, 1:1000), total PKCα (1:1000, all Cell Signaling Technology), TNFR1 (1:1000), TNFR2 (1:1000, Santa Cruz Biotechnology, Dallas, TX), c-myc (1:2000) and cyclin D1 (1:2000, both ProteinTech Group, Chicago, IL). GAPDH antibody was a control (1:2000, Hangzhou Goodhere Biotech, China).

### Detection of nascent protein synthesis

HeLa cells were transfected with negative control siRNA (si-NC) or si-PGRN and incubated for 48 h or were treated with DMSO or 100 μM rapamycin for 1 h followed by PBS or 500 ng/mL rhPGRN for 6 h. Nascent protein synthesis was accessed by use of the Click-iT Plus O-propargyl-puromycin (OPP) Protein Synthesis Assay Kit (Life Technologies Corp., Eugene, OR). The cells were incubated in medium with 20 μM Click-iTOPP working solution for 30 min, then fixed with 3.7% formaldehyde in PBS and permeabilized with 0.5% Triton X-100. Cells were stained with Click-iT Plus OPP reaction cocktail containing Alexa Fluor 488 picolyl azide and imaged by fluorescence microscopy. Quantification of OPP labeling was as described [49].

### Co-immunoprecipitation (Co-IP) assay

Approximately 500 μg of cellular extract prepared from rhPGRN-pretreated HeLa cells was incubated with anti-PGRN or control IgG (25 μg/mL) antibodies for 1 hr, followed by incubation with 30 μL of protein A/G-agarose at 4°C overnight. Bound protein was examined by Western blot with anti-TNFR1 and anti-TNFR2 antibodies.

### Cell transformation and proliferation assays

For colony formation assay in soft agar culture, 2 mL of 0.6% agar in DMEM was added to 6-well culture plates and allowed to solidify over 2 h. An amount of 0.5 mL of 0.3% agar in DMEM with 1×10^5^ H8 cells per plate was added above this layer. When the top agar layer was solidified, 0.3 mL DMEM containing PBS or 500 ng/mL rhPGRN with or without 100 μM rapamycin was added and replaced every 3 days. Plates were incubated for 4 weeks until the clones were counted. For cell proliferation assay, H8, SiHa or HeLa cells were seeded 2×10^3^ per well in 96-well plates followed by the addition of PBS or 500 ng/mL rhPGRN with or without 100 μM rapamycin. At days 0-4, cell proliferation was accessed by use of the Cell Counting Kit-8 (CCK-8; Dojindo Laboratories, Tokyo) and cell number counting assay as described [48].

### Cell survival assays

For colony formation assay, H8 cells were seeded 1 ×10^4^ per well in 6-well plates in medium containing 1% FBS with the indicated treatment and incubated for 2 weeks, then clones were counted after fixing and staining with 0.5% crystal violet solution. For anoikis assay, HeLa cells (1×10^5^) were resuspended in 1 mL serum-free DMEM containing PBS or 500 ng/mL rhPGRN with or without 100 μM rapamycin and grown in polypropylene tubes at 37°C, 5% CO_2_. After 48 h, 20 μL from each suspension was mixed with 20 μL trypan blue staining solution (Beyotime Institute of Biotechnology, China) and counted in a hemocytometer. The number of unstained living cells and heavily stained dead cells was recorded.

### Cell motility and migration assays

H8 cell monolayers were wounded with use of a plastic tip. Then cells were replaced with serum-free DMEM and treated with DMSO or 100 μM rapamycin 1 h before treatment with PBS or 500 ng/mL rhPGRN. The motility of cells was photographed under a light microscope at days 0, 1 and 2. For transwell migration assay, 2 × 10^4^ H8 cells suspended in serum-free DMEM with or without 500 ng/mL rhPGRN in the presence or absence of 100 μM rapamycin were added to transwell inserts (8-μm pore size; Millipore, Billerica, MA), held in 24-well companion plates with DMEM containing 10% FBS, and incubated 36 h. Migrated cells at the bottom of the filter were photographed under a light microscope after fixing and visualizing by Giemsa staining. The number of migrated cells in each chamber was quantified by counting five fields under 20 × magnification.

### BrdU incorporation assay

HeLa cells were treated with DMSO or 100 μM rapamycin for 1 h, then with PBS or 500 ng/mL rhPGRN for 6 h, and BrdU labeling was assessed by use of the FITC BrdU Flow Kit (BD Biosciences, San Diego, CA). Flow cytometric analysis was performed using a Coulter cytomics FC500 flow cytometer (Beckman Coulter, Fullerton, CA) with CXP software (Beckman Coulter).

### Xenograft tumor studies

The experimental protocols were approved by the Institutional Animal Care and Use Committee of Shandong University [no. 201402062]. The investigation conformed to the US National Institutes of Health Guide for the Care and Use of Laboratory Animals and was performed in accordance with the ARRIVE guidelines (http://www.nc3rs.org/ARRIVE). All mice were housed under specific pathogen-free conditions and maintained on a 12-h light/dark cycle at 25±2°C, with free access to food and water. Six-week-old female BALB/c nude mice (nu/nu) were purchased from Vital River Laboratories (Bejing) and acclimated to housing conditions for at least 1 week before experiments.

We subcutaneously injected mice with 1 × 10^7^ H8 or HeLa cells, then on days 6 after injection, cell-implanted mice implanted were divided into 4 groups (n=8 each) for intraperitoneal injection of rhPGRN (10 mg/kg body weight) or PBS and rapamycin (1.5 mg/kg body weight) or DMSO every 3 days. Tumor size was measured every 3 days by use of calipers and calculated as V (mm^3^) =0.5×ab^2^, where a and b represent the long and perpendicular short diameter (mm) of the tumor, respectively. At the end of experiments, mice were killed by cervical dislocation under sodium pentobarbital anesthesia (50 mg/kg) and tumors were excised and weighed.

### Statistical analysis

Data are expressed as mean ± SD or SEM. Differences were estimated by one-way ANOVA followed by Duncan's multiple range test. *P*<0.05 was considered statistically significant. Correlation of PGRN expression with phospho-mTOR-Ser2448 expression in cervical cancer tissues was analyzed by Pearson correlation test.

## SUPPLEMENTARY FIGURES


