# Supplementary material for: Growth factor progranulin promotes tumorigenesis of cervical cancer via PI3K/Akt/mTOR signaling pathway
Source: Oncotarget. 2016 Aug 9;7(36):58381–95. doi: 10.18632/oncotarget.11126 (PMC5295437; doi:10.18632/oncotarget.11126)
Supplement: Supplementary file 1 [file oncotarget-07-58381-s001.pdf]

Growth factor progranulin promotes tumorigenesis of cervical cancer via PI3K/Akt/mTOR signaling pathway

SUPPLEMENTARY FIGURES

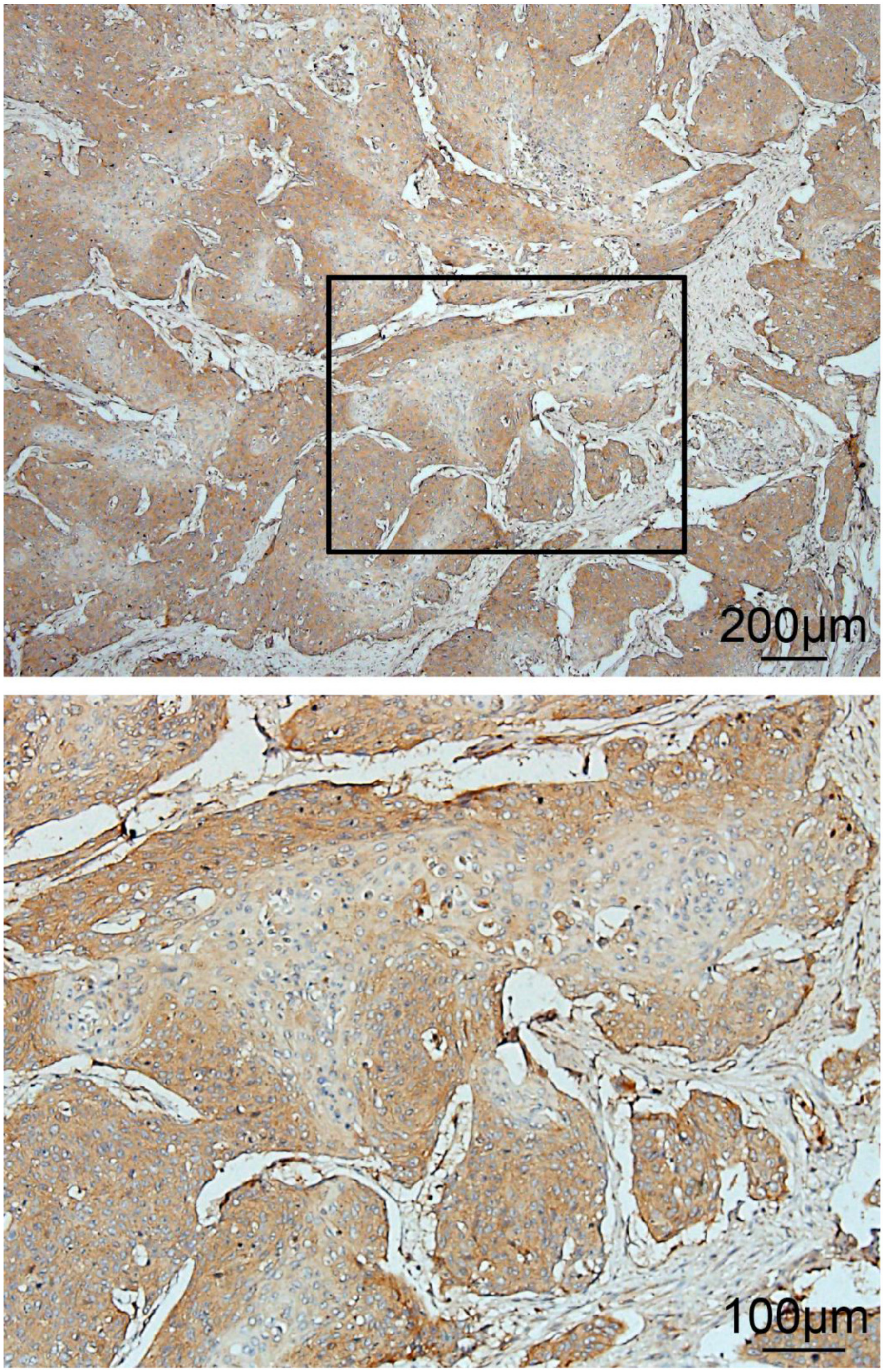

Supplementary Figure S1: Representative photomicrographs of PGRN immunohistochemical staining in cervical cancer tissues showed diverse expression of PGRN in the center and edge of tumor nests.

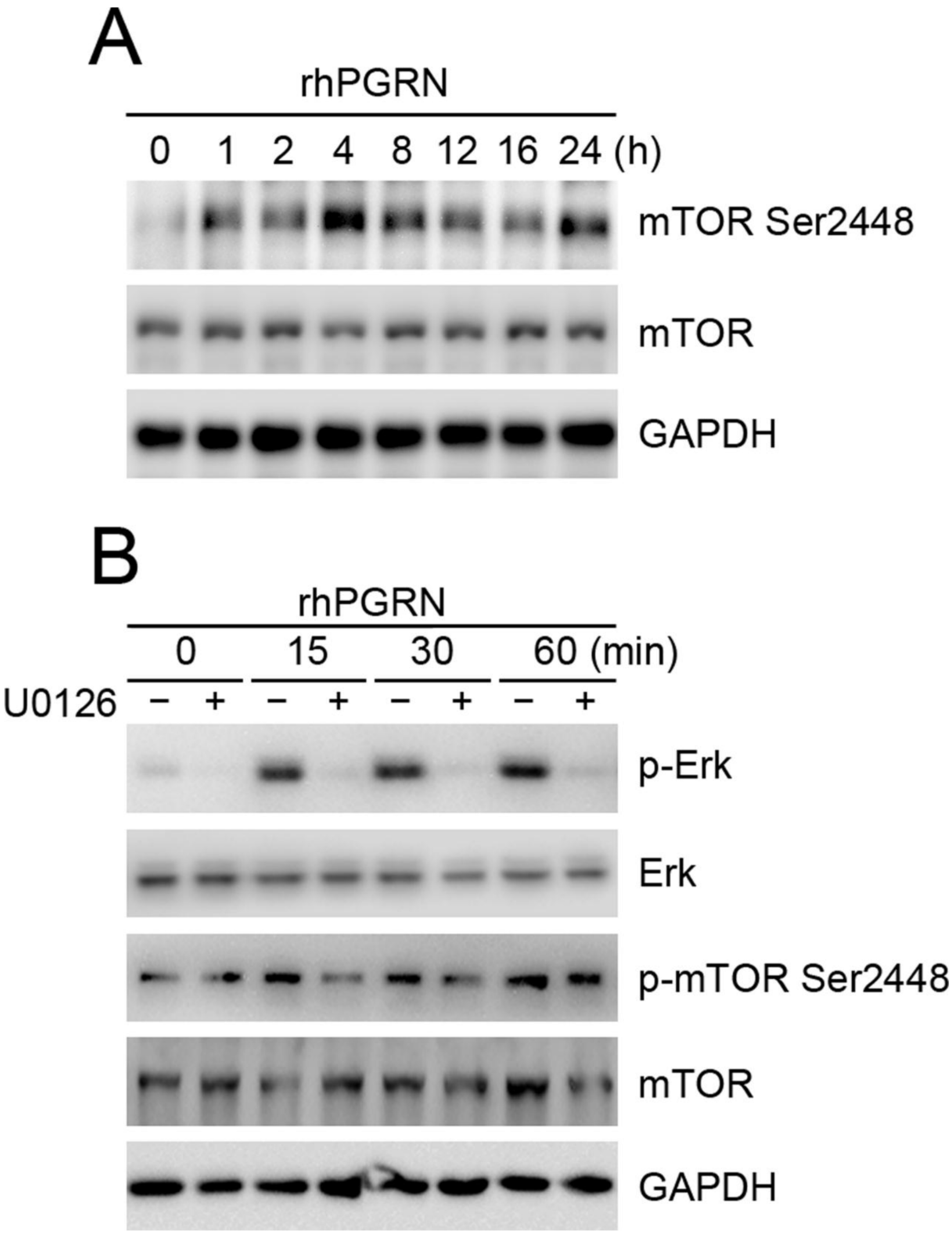

Supplementary Figure S2: A. Western blot assay of phospho-mTOR-Ser2448 in HeLa cells treated with 500 ng/mL rhPGRN at longer periods. **B.** Western blot assay of phospho-mTOR-Ser2448 in DMSO- or U0126-pretreated HeLa cells at 15, 30 and 60 min after 500 ng/mL rhPGRN stimulation.
